# Molecular signatures of mammalian hibernation: comparisons with alternative phenotypes

**DOI:** 10.1186/1471-2164-14-567

**Published:** 2013-08-20

**Authors:** Yichi Xu, Chunxuan Shao, Vadim B Fedorov, Anna V Goropashnaya, Brian M Barnes, Jun Yan

**Affiliations:** 1 CAS-MPG Partner Institute for Computational Biology, Shanghai Institutes of Biological Sciences, Chinese Academy of Sciences, 320 Yue Yang Road, Shanghai 200031, China; 2 Institute of Arctic Biology, University of Alaska Fairbanks, Fairbanks, AK 99775, USA

**Keywords:** Hibernation, Microarray, Molecular signatures, Liver, *Urocitellus parryii*

## Abstract

**Background:**

Mammalian hibernators display phenotypes similar to physiological responses to calorie restriction and fasting, sleep, cold exposure, and ischemia-reperfusion in non-hibernating species. Whether biochemical changes evident during hibernation have parallels in non-hibernating systems on molecular and genetic levels is unclear.

**Results:**

We identified the molecular signatures of torpor and arousal episodes during hibernation using a custom-designed microarray for the Arctic ground squirrel (*Urocitellus parryii*) and compared them with molecular signatures of selected mouse phenotypes. Our results indicate that differential gene expression related to metabolism during hibernation is associated with that during calorie restriction and that the nuclear receptor protein PPARα is potentially crucial for metabolic remodeling in torpor. Sleep-wake cycle-related and temperature response genes follow the same expression changes as during the torpor-arousal cycle. Increased fatty acid metabolism occurs during hibernation but not during ischemia-reperfusion injury in mice and, thus, might contribute to protection against ischemia-reperfusion during hibernation.

**Conclusions:**

In this study, we systematically compared hibernation with alternative phenotypes to reveal novel mechanisms that might be used therapeutically in human pathological conditions.

## Background

Hibernation in mammals is a naturally occurring process of profound suppression of metabolic demand and tolerance to hypothermia that enables individuals to fast for 6–8 months. In addition, animals are protected from injury following cycles of hypoperfusion and reperfusion that occur as they cool and rewarm. In an extreme example of hibernation, the Arctic ground squirrel (AGS, *Urocitellus parryii,* family *Sciuridae*) decreases its basal metabolism by 98% and body temperature (Tb) to as low as -2.9°C when it enters bouts of torpor, which can last for over 3 weeks [1]. In all small mammalian hibernators, torpor is periodically interrupted by spontaneous arousal episodes when animals raise their metabolism and Tb returns to normal, euthermic levels for no more than 24 h. Animals then re-enter torpor and repeat the torpor-arousal cycle until the end of the 6–8-month hibernation season. Gene transcription [2] and translation [3] are dramatically suppressed during torpor, and many additional physiological parameters also greatly decrease and then recover after arousal, e.g. heart rate, respiration, electrical activity, and renal function [4].

The complex phenotypes in hibernation are examples of extreme physiology that represent limits to adaptive design and are therefore intriguing to biologists. Fundamental questions surrounding hibernation remain largely unanswered; for instance, the relationship between torpor and arousal and wake and sleep. Electroencephalogram (EEG) recordings during entry into torpor suggest that torpor and sleep may be homologous [5]. During deep torpor, however, the EEG is isoelectric, and it has been hypothesized that the associated low brain temperature is incompatible with the restorative function of sleep [6]. EEG recordings in the AGS taken during the arousal cycle have demonstrated that the majority of time at high body temperatures is spent in slow-wave and rapid eye movement sleep, which may represent compensation for the accumulation of sleep debt during torpor [7]. Arousal episodes also restore metabolic homeostasis lost from the plasma during torpor [8].

The role of circadian rhythms in hibernation is also unclear. Core clock genes including *Per1*, *Per2*, and *Bmal1* stop oscillating in their expression levels during torpor in European hamsters (*Cricetus cricetus L.*), which suggests that the circadian clock is arrested during hibernation [9]. Circadian rhythms of body temperature have not been detected during hibernation in either Arctic or European ground squirrels, and arrhythmic patterns of body temperature continue for several weeks after the animals end torpor and resume constant high body temperatures in spring, provided they experience constant darkness [10,11]. However, very low amplitude cycles of body temperature persist during torpor in the golden-mantled ground squirrel (*Callospermophilus lateralis*) [5].

More information about the molecular basis of the phenotypes displayed during mammalian hibernation may lead to the development of novel clinical approaches for the treatment of injuries and disease in humans. For example, the tissues of hibernators experience regular cycles of rapid changes in blood pressure and flow during the torpor-arousal cycle; yet organs such as the liver, brain, heart, and gut do not exhibit the type of damage expected from ischemia-reperfusion in non-hibernating animals, even when hibernators experience long periods of no blood flow, blood loss, or trauma [4,12-15]. An understanding of the molecular mechanisms of natural resistance to hypoperfusion-reperfusion in hibernators may result in treatments for or the prevention of heart attack, stroke, or perioperative injuries. This approach could be extended to preserve transplant organs, prevent disuse atrophy, or treat sleeping disorders.

Here, we address these questions by studying the molecular signatures of hibernation in mammals. Microarray experiments generate differential molecular signatures under physiological and biochemical perturbations that reflect genetic regulatory mechanisms [16]. Our initial high-throughput efforts to create molecular signatures of hibernation in small mammalians had limited coverage that was insufficient for comprehensive comparisons to signatures that define potentially related regulatory pathways in model organisms [17,18]. In this study, we designed a new cDNA microarray for the Arctic ground squirrel with genome-wide coverage. Using this microarray, we identified wide molecular signatures of hibernation in the AGS liver during different stages of the torpor-arousal cycle. Gene expression changes in the liver are related to the overall inhibition of metabolism, mechanisms of differential fuel use, and long-term fasting during hibernation. In this systematic dissection of the hibernation phenotype, we compared hibernation signatures with those from different physiological conditions in mice, including calorie restriction, PPARα knockout, sleep deprivation, cold exposure, and ischemia-reperfusion (Table 1). Our study is the first attempt to investigate the relationship between hibernation and other physiological conditions at the molecular level.

**Table 1:** Mouse microarray datasets on hibernation-related physiological conditions collected from GEO or ArrayExpress databases

**GEO/ArrayExpress ID**	**Stress**	**Source**	**Microarray**
E-MEXP-748	Calorie restriction	Liver	Mouse4302
GSE1093	Calorie restriction	Liver	MG-U74A
GSE2431	Calorie restriction	Liver	MG-U74A
GSE10657	Ischemia-reperfusion	Liver	Mouse4302
GSE9441	Sleep deprivation	Liver	Mouse4302
GSE8292	PPARα knockout	Liver	Mouse4302
GSE20645	Cold exposure	Oligodendrocyte precursor cells	Mouse4302

## Methods

### Animals

AGS tissue samples were taken from the same animals as in our previous microarray study [17]. Briefly, AGS were trapped during July near Toolik Lake in northern Alaska (68°N 149°W, elevation 809 m) and transported to the University of Alaska, Fairbanks. Animals were initially housed at 20±2°C with a 16:8-h light:dark photoperiod and provided with Mazuri Rodent Chow and water *ad libitum*. Hibernating animals were previously implanted abdominally with temperature-sensitive radio transmitters and maintained at a 4:20-h photoperiod. Core Tb was monitored for precise stage of torpor and arousal by an automated telemetry system that measures and records core Tb every 10 min [19]. Animals sampled in torpor and arousal episodes were deprived of food for the entire period of hibernation. Animals were collected early in a torpor bout after 10–20% of the duration of the bout (early torpor, between 4–6 days, n = 4). Animals late in a torpor bout were collected after 80–90% of the duration of the previous bout (late torpor, between 8–12 days, n = 5). Animals sampled early after spontaneously arousing from torpor were collected 1–2 h after Tb had increased above 30°C during rewarming (early arousal, n = 4). Animals late in the arousal episode were collected 7–8 h after Tb had increased above 30°C (late arousal, n = 4). Post-reproductive euthermic animals (P, n = 7) that had completed reproductive maturation and regression as assessed by external inspection of gonads and genitalia and had entered molt were used as a non-hibernating control. Torpid animals were euthanized by decapitation without anesthesia. Aroused and post-reproductive animals were deeply anesthetized with isoflurane vapors and decapitated. Animal protocols were approved by the University of Alaska, Fairbanks, Institutional Animal Care and Use Committee.

### AGS cDNA libraries construction

We constructed cDNA libraries enriched for full-length inserts by applying the SMART template-switching protocol, primer extension PCR [20], normalization, and subtraction [21], as described by Federov et al. [22]. Five AGS cDNA libraries were constructed: 100 - mixed library (brain, liver, heart, brown adipose tissue, and muscle) used for subtracting all other libraries, 101 - heart, 102 - brain, and 103, 104 - liver. 24,371 AGS ESTs (expressed sequence tags) 496 (SD ±133) bases long were sequenced at the University of Alaska, Fairbanks AGS cDNA library project. Equal amounts of mRNA from hibernating and non-hibernating animals were pooled in cDNA libraries to enhance transcript representation.

### AGS EST clustering and annotation

We developed a bioinformatics pipeline to cluster and annotate 24,371 AGS ESTs. The 1.90X coverage assembly of the 13-lined ground squirrel (*Ictidomys tridecemlineatus*) genome (speTri1, Jun 2006) was obtained from Ensembl, including genome sequences and 14,831 gene annotations. The low coverage of the genome may still lead to incomplete gene models and annotations. EST sequences of the golden-mantled (*Callospermophilus lateralis*; 8,803 sequences) and 13-lined ground squirrels (5,256 sequences) were obtained from NCBI. We aligned the AGS ESTs and those of the other two squirrels to speTri1 with BLASTN, requiring > 50% alignment length and > 95% identity. The sim4 program was used to identify the accurate splice sites on the ESTs. Meanwhile, we aligned the human and mouse RefSeq mRNA sequences to speTri1 using the same method, requiring > 50% alignment length and > 85% identity. The aligned ESTs and RefSeqs were clustered if they shared common splice sites or their alignments overlapped by > 50% of the length of the shorter sequence. The unaligned ESTs were aligned and clustered to each other. To remove the redundancy on EST clusters due to genome duplication or incomplete assembly, two or more EST clusters were merged into one if they shared > 80% of the ESTs in each cluster.

We chose the annotation for each EST cluster in the following order of precedence: speTri1 annotation, human RefSeq, and mouse RefSeq. For EST clusters that were not annotated by the above, all ESTs in the cluster were patched together to obtain an annotation in the NR (non-redundant) database by BLASTX. Overall, 64% of AGS ESTs were reliably aligned to speTri1 and 54% were annotated with known gene symbols. 9,600 cDNA clones were printed on the membrane cDNA arrays.

### AGS cDNA microarray hybridization

Total RNA was extracted from tissues with RNeasy Kits (Qiagen) and stored at -80°C, linearly amplified by the Illumina TotalPrep RNA Amplification Kit (Ambion), labeled with [33P]dCTP, and hybridized with array filters as previously described [22]. All RNA samples were processed in the same batch. The filters were exposed to phosphorimager screens for 4 days and scanned at 50 μm resolution in a Storm Phosphorimager. Images were analyzed by the ImaGene program (Biodiscovery).

### AGS cDNA microarray analysis

Out of 9,600 probes, 6–8,000 on the cDNA microarray had a signal/background > 2. Only 50–120 probes were undetected. The signals were subtracted from the background, divided by the median, and log2 transformed. Early arousal and late arousal were treated together as arousal (A). Early torpor and late torpor were treated together as torpor (T). In total, A, T, and P contained eight, nine, and seven animal replicates, respectively. Three-state (A, T, P) one-way ANOVA was performed on 9,600 probes. 5,314 unique genes with human symbols were identified after removing redundancy at the probe level according to the minimal ANOVA p-value. False discovery rate (FDR) was evaluated by q-value using a conservative method proposed by Storey and Tibshirani [23]. Differentially expressed genes between each pair of two states were selected by ANOVA models with q-values < 0.05 and Tukey’s honestly significant difference (HSD) test p-values < 0.05. Data analysis was performed in R 2.11.1. Microarray data series were submitted to the National Center for Biotechnology Information Gene Expression Omnibus (NCBI GEO) under the accession number GSE38700.

### Mouse microarray analysis

Normalized data from GSE1093, GSE2431, and raw data from the other microarrays were either collected from EBI ArrayExpress repository or NCBI GEO datasets (Table 1). Raw data were normalized by robust multi-array average in the R package affy. The Mouse4302 microarray was annotated by the R package mouse4302. The MG-U74A microarray was annotated by Affymetrix platform GPL81 on NCBI. Mouse gene symbols in annotation were replaced by the gene symbols of human homologues, which facilitated the comparison of AGS and mouse microarrays. According to the experimental design, ANOVA (for PPARα knockout and sleep deprivation) and t-tests (for cold exposure) were performed to obtain differentially expressed genes at the q < 0.05 significance level. Differentially expressed genes in PPARα knockout were also limited by a threshold on log2 fold-change as suggested by Rakhshandehroo et al. [24]. Differentially expressed genes in calorie restriction were determined by a meta-analysis strategy [25], i.e. requiring consistent changes of gene expression in three out of four experiments. To remove redundancy on multiple probes annotated by the same gene, we kept the probe with the minimum q-value. Data analysis was performed in R 2.11.1.

### Comparison of AGS and mouse microarrays

To determine the associations between hibernation and calorie restriction, PPARα knockout, sleep deprivation, and cold exposure, we calculated Pearson’s correlation coefficient (PCC) of log2 fold-changes of differentially expressed genes in both non-hibernating physiological conditions and hibernation. The p-value for testing the null hypothesis (PCC = 0) was calculated based on Pearson's product moment correlation coefficient. To compare ischemia-reperfusion to hibernation, we turned to the patterns of gene expression, assuming that P, T, and A in hibernation are similar to the control (CN), ischemia (IS), and reperfusion (RP), respectively. A mathematical clustering technique, self-organizing map, was applied to cluster the patterns of gene expression with robustness and accuracy [26]. Data analysis was performed in R 2.11.1.

### Comparison of AGS microarrays and PPARα and *CIRBP* target genes

We required the target PPARα genes to be differentially expressed in PPARα knockout and bound by PPARα. The PPARα binding sites were collected from chromatin immunoprecipitation with massively parallel DNA sequencing (ChIP-Seq) [27]. Differentially expressed genes in PPARα knockout [24] were searched for PPARα bound from 10 kb upstream to 3 kb downstream, relative to the start of transcription, resulting in 614 target genes for PPARα.

We required the *CIRBP* (cold-inducible RNA-binding protein) target genes to be differentially expressed in *CIRBP* knockdown and bound by *CIRBP* clusters [28]. The binding *CIRBP* clusters were collected from cross-linking and immunoprecipitation-high-throughput sequencing (CLIP-Seq) [28]. Two hundred and one target genes were obtained for *CIRBP*. Fisher’s exact test was performed to detect the enrichment of target genes in differentially expressed genes during hibernation.

## Results

### Identification of molecular signatures of hibernation

We analyzed differential gene expression in AGS as a model species for mammalian hibernation. The custom microarray comprised 6,403 annotated AGS genes, among which 5,314 were annotated with human gene symbols. Because the aim of this study was to compare AGS with non-hibernating model animals, we focused on these 5,314 genes in downstream analyses.

**Figure 1:**
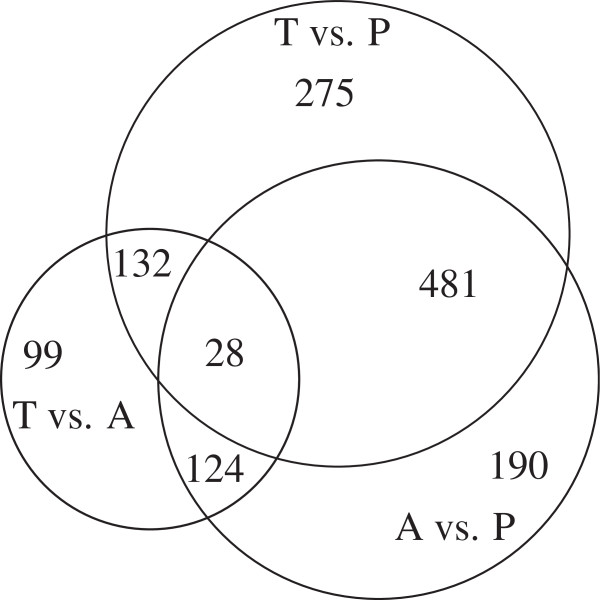
**Venn diagram of differentially expressed genes during hibernation.** 916, 823, and 383 genes were differentially expressed in T vs. P, A vs. P, and T vs. A comparisons, respectively. T: torpor; A: arousal episodes; P: post-reproduction.

More than one thousand genes (Additional file 1) exhibited differential expression (q-value < 0.05) across torpor (T), arousal episodes (A), and post-reproduction (P). Of these, 916, 823, and 383 genes were significantly differentially expressed in T vs. P, A vs. P, and T vs. A comparisons (Tukey’s HSD test p < 0.05), and were labeled TP, AP, and TA signatures, respectively. TP and AP signatures have 509 genes in common (Figure 1). Of these genes, 508 that had consistent changes of direction in T vs. P and A vs. P were defined as hibernation signatures. This indicates that the seasonal differences in mRNA levels between hibernating and post-reproduction states (T vs. P or A vs. P) are larger than those within hibernating states (A vs. T). Gene set enrichment analysis [16] was performed to calculate the enrichment of canonical pathways in the differentially expressed genes (Table 2).

**Table 2:** Top ranking enriched gene sets in different signature groups during hibernation

**Gene set description**	**# Genes in set**	**# Overlapping genes**	**FDR q-value**
477 genes that were over-expressed during T compared to P			
Lipid and lipoprotein metabolism	478	26	3.5×10^-9^
Fatty acid metabolism	42	9	1.8×10^-7^
Valine, leucine, and isoleucine degradation	44	9	1.8×10^-7^
Fatty acid, triacylglycerol, and ketone body metabolism	168	14	4.9×10^-7^
PPAR signaling pathway	69	9	7.1×10^-6^
439 genes that were under-expressed during T compared to P			
Lipid and lipoprotein metabolism	478	34	0
Biological oxidations	139	19	0
Drug metabolism - cytochrome P450	72	16	0
Phase II conjugation	70	15	0
301 genes that were over-expressed during A compared to P			
Lipid and lipoprotein metabolism	478	14	2.7×10^-3^
522 genes that were under-expressed during A compared to P			
Lipid and lipoprotein metabolism	478	36	0
Biological oxidations	139	29	0
Retinol metabolism	64	16	0
Phase II conjugation	70	19	0
Steroid hormone biosynthesis	55	16	0
291 genes that were over-expressed during T compared to A			
RNA metabolism	330	23	0
Ribosomes	88	16	0
60S ribosomal subunit, cytoplasmic	47	13	0
92 genes that were under-expressed during T compared to A			
HCF-1 complex	19	3	8.4×10^-3^

Gene set enrichment analysis (v3.87) was performed with canonical gene pathways in the comparisons between torpor (T), arousal episodes (A), and post-reproduction (P).

### Comparison of the calorie restriction and hibernation datasets

During hibernation, fasting can last for most of the year; for example, 7–8 months in female Arctic ground squirrels [29]. To examine the relationship between changes in gene expression during hibernation and those during the best available animal model of calorie restriction, we collected calorie restriction (10–40% calories) microarray datasets from mice [GSE1093, GSE2431, and E-MEXP748] [30-32]. This included two short-term (3 or 8 weeks) and two long-term experiments (17 or 24 months). We focused on the 253 calorie restriction affected genes (Additional file 2) that consistently showed significant differential expression in at least three out of four experiments.

Because the AGS fasts during hibernation, calorie restriction affected genes were compared with TP, AP, and hibernation signatures (Table 2). These genes overlapped best with hibernation signatures (PCC = 0.58, p = 0.01). Eighteen genes were both significant in calorie restriction and hibernation signatures (Additional file 3). The over-expression of genes involved in gluconeogenesis (*PCK1*) and the under-expression of genes in triglyceride biosynthesis (*ELOVL6*, *LPIN2*) were observed during both hibernation and calorie restriction. In particular, Lipin 2 (*LPIN2*), a phosphatidate phosphatase involved in triglyceride metabolism and implicated in adipocyte development was under-expressed [33]. In addition to these metabolic genes, we identified that a transmembrane receptor, growth hormone receptor (*GHR*), was under-expressed during both hibernation and calorie restriction. *GHR* expression in the liver decreases after food restriction [34]; its under-expression may play a regulatory role in slowing down cell growth when energy is conserved.

### Comparison of the PPARα knockout and hibernation datasets

PPARα is an important transcription factor regulating fatty acid metabolism [35]. To examine if PPARα is associated with the shift from the metabolism of glucose to fatty acids in hibernation, we analyzed a PPARα knockout microarray dataset [GSE8292] [24], consisting of wild type mice and PPARα gene knockout mice under two feeding conditions (with and without PPARα agonists). Two-way ANOVA was performed with mouse genotype and feeding condition as factors. Of 21,248 genes, expression levels of 1,587 differed significantly in PPARα knockout (q-value < 0.05, log2 fold-change > 0.5).

TP signatures were significantly negatively correlated with PPARα knockout (PCC = -0.19, p = 0.036, Table 3). One hundred and twenty-three genes were both differentially expressed in PPARα knockout and torpor (Additional file 3). Of these genes, 33 were over-expressed in PPARα knockout and torpor, and were enriched in mitochondrial fatty acid β oxidation (*HADHB*, *ACADL*, and *ACADVL*) and PPAR signaling (*EHHADH*, *ACADL*, *ACOX1*, *HMGCS2*, and *SLC27A2*). *HMGCS2*, a key gene involved in ketogenesis, is regulated by PPARα. Thirty-eight genes were under-expressed in PPARα knockout and torpor, but were enriched in cholesterol and steroid biosynthesis (*FDFT1*, *FDPS*, *HMGCR*, and *SC4MOL*). Thus, PPARα plays a dual role of up-regulating fatty acid catabolism and ketone body synthesis while down-regulating cholesterol and steroid biosynthesis, which are energetically costly. Comparison of the PPARα knockout and torpor datasets indicates that the genes regulated by PPARα are involved in metabolic shifts during torpor.

**Table 3:** Hibernation signatures, TP signatures, and AP signatures compared with calorie restriction and PPARα knockout

		**TP signatures (916 genes)**	**AP signatures (823 genes)**	**Hibernation signatures (508 genes)**
Calorie restriction (253 genes)	Overlap #	24	29	18
	PCC	0.41	0.23	0.58
	p-value	0.04	0.23	0.01
PPARα knockout (1587 genes)	Overlap #	123	103	73
	PCC	−0.19	−0.14	−0.15
	p-value	0.04	0.17	0.19

Overlap # is the number of overlapping genes. PCC is Pearson’s correlation coefficient of log2 fold-changes of overlapping genes. P-value is calculated based on Pearson's product moment correlation coefficient to test the null hypothesis (PCC = 0). TP: torpor vs. post-reproduction; AP: arousal episodes vs. post-reproduction.

To further study the regulatory function of PPARα during hibernation, we collected 614 PPARα target genes by combining ChIP-Seq [27] and differentially expressed genes in PPARα knockout. Out of 916 genes in the TP signatures, 65 were PPARα target genes (Fisher’s exact test p = 0.002, odds ratio = 1.6, Additional file 3).

### Comparison of the sleep deprivation and hibernation datasets

We collected a sleep deprivation dataset [GSE9441] [36] consisting of three strains of mice in two sleep states (sleeping control and sleep deprived for 6 h). Two-way ANOVA was performed with mouse strain and sleep state as factors. Out of 21,248 genes, we identified 1,156 that consistently showed significantly different expression levels during sleep deprivation among the mouse strains (q-value < 0.05).

To determine how sleep deprivation is related to torpor or arousal, we compared gene expression changes in sleep deprivation with TA signatures. Thirty-seven out of 383 genes in the TA signatures exhibited similar differential expression patterns during sleep deprivation (Additional file 3). PCC of log2 fold-changes of 37 genes was ˗0.66 (p = 10^-6^, Table 4), which indicates that, in terms of global gene expression in the liver, the effect of sleep is more similar to torpor, whereas sleep deprivation is similar to arousal. In contrast, sleep deprivation and TP signatures were not significantly correlated. Next, we focused on the genes with the opposite changes between sleep deprivation and TA signatures. Twelve genes were over-expressed during sleep deprivation and arousal episodes; these included four heat shock protein genes (*HSPH1*, *HSP90AA1*, *HSPA8*, and *HSP90AB1*). *CIRBP*, in response to low temperature stimulus, was the most significantly under-expressed gene out of 16 during sleep deprivation and arousal episodes. Temperature-responsive genes were noticeably similar in expression changes during both mouse sleep and AGS torpor.

**Table 4:** TP signatures and TA signatures compared with sleep deprivation and cold exposure

		**TP signatures (916 genes)**	**TA signatures (383 genes)**	**Consistent TP-TA (144 genes)**
Sleep deprivation (1156 genes)	Overlap #	82	37	/
	PCC	0.19	−0.66	/
	p-value	0.08	7.6×10^-6^	/
Cold exposure (1675 genes)	Overlap #	106	41	19
	PCC	−0.002	0.02	0.49
	p-value	0.98	0.9	0.03

Overlap # is the number of overlapping genes. PCC is Pearson’s correlation coefficient of log2 fold-changes of overlapping genes. P-value is calculated based on Pearson's product moment correlation coefficient to test the null hypothesis (PCC = 0). Consistent TP-TA is the consistent part of TP and TA signatures (hypothermia signatures). TP: torpor vs. post-reproduction; TA: torpor vs. arousal episodes.

### Comparison of the cold exposure and hibernation datasets

To further examine the relationship between temperature response and hibernation, we obtained a mouse cold exposure dataset [GSE20645] [37], consisting of two groups (n = 4) of mouse oligodendrocyte precursor cells cultured at 31.5°C and 37°C, for 48 h. Although this system is quite different from the AGS liver, the influence of a temperature decrease is the most common feature of the two datasets. We considered 31.5°C cold exposure and 37°C the control. Of 21,248 genes, 1,675 were differentially expressed during cold exposure (q-value < 0.05).

AGS body temperature is much lower during torpor than arousal or post-reproduction. Thus, we compared differentially expressed genes during cold exposure with TP and TA signatures (Table 4). The PCC of log2 fold-changes of overlapping genes in cold exposure was not significant for either TP or TA signatures. However, genes with a consistent change in direction common to both TP and TA signatures were significantly positively correlated with cold exposure (PCC = 0.49, p = 0.03, Table 4). Nineteen genes were significant both in cold exposure and the consistent signatures of TP and TA (Additional file 3). Of them, *CIRBP* displayed the most significant change. The heat shock protein *HSPH1* and the *HSPH90* co-chaperone, *CHORDC1*[38], both decreased during cold exposure and torpor. Therefore, gene expression changes common to TP and TA signatures are also common to cold-induced gene expression changes and are defined as hypothermia signatures.

### Comparison of the circadian and hibernation datasets

**Figure 2:**
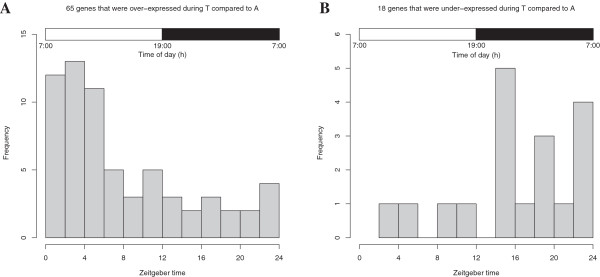
**Distribution of circadian phases of differentially expressed genes in torpor vs. arousal episodes.** Open and filled bars symbolize light (mice are asleep) and dark (mice are active) phases. Circadian peaks of genes that were over-expressed in torpor appeared mostly in the light phase **(A)** and circadian peaks of genes that were under-expressed in torpor appeared mostly in the dark phase **(B)**. T: torpor; A: arousal episodes.

We collected 2,276 circadian oscillating genes from multiple microarray studies in mouse livers, and integrated their circadian peak and trough information [39]. Eighty-three circadian genes were differentially expressed in TA signatures (Additional file 3). Interestingly, the circadian peaks of 65 genes over-expressed during torpor appeared mostly during the light phase (Zeitgeber Time, ZT0-ZT12), when the mice were asleep. The circadian peaks of 18 genes under-expressed during torpor appeared mostly during the dark phase (ZT12-ZT24) when the mice were awake (Figure 2). Fisher’s exact test (p = 10^-5^, odds ratio = 10.4) shows a strong association between genes over-expressed during torpor and those over-expressed during sleep. This is consistent with the results of our comparison between sleep deprivation and hibernation. The daily sleep-wake cycle in mice shares significant molecular signatures with the AGS torpor-arousal cycle during hibernation, with torpor being similar to sleep.

*CIRBP* emerges as one of the most significant genes common to TA signatures and circadian genes that peak during the light phase. *CIRBP*, also over-expressed during cold exposure and under-expressed during sleep deprivation, is a major cold shock protein in mammals involved in the modulation of transcription and translation [40]. A recent study reported that *CIRBP* plays an important role in post-transcriptional regulation of the circadian clock [28]. To examine its role in torpor, we collected 201 *CIRBP* target genes determined by CLIP-Seq and their differential expression upon *CIRBP* knockdown (q < 0.05) [28]. Hypothermia signatures were enriched within the *CIRBP* target genes (Fisher’s exact test p = 0.01, odds ratio = 3.5). These target genes include *DERL2*, *FUBP1*, *NNT*, *SMAD5*, *TARDBP*, and *THRA*, consisting mainly of transcription factors and RNA-binding proteins. Notably, *TARDBP*[41], under-expressed in both TP and TA signatures, was over-expressed during sleep deprivation and showed a circadian oscillation peak in the dark phase (ZT17).

### Comparison of the ischemia-reperfusion and hibernation datasets

We obtained a mouse liver ischemia-reperfusion dataset [GSE10657] [42] including five states (control, ischemia 30 min, ischemia 60 min, ischemia 90 min, and reperfusion 1 h) in young and old mice. Old mice experienced more severe injuries than young mice. Ischemia 60 min showed the smallest correlation with the control in gene expression. Thus, we chose ischemia 60 min in old mice to represent ischemia (IS), combined with the control (CN) and reperfusion (RP). For comparisons, we considered T, with its conditions of low blood pressure and hypoperfusion, as the counterpart of ischemia, A as the counterpart of reperfusion, and P as the counterpart of the control during hibernation.

**Figure 3:**
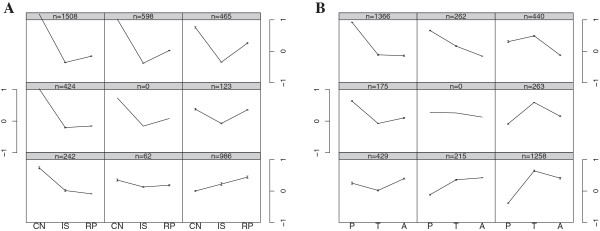
**Patterns of gene expression in ischemia-reperfusion (CN-IS-RP) and hibernation (P-T-A).** The expression values were normalized to (-1,1) in the ischemia-reperfusion **(A)** and hibernation datasets **(B)**. Gene expression trends were clustered into nine patterns by self-organizing mapping. The numbers of genes in each pattern are shown on the top of the grids. The patterns that contain the most genes are in the top left and bottom right of each map. One of the major patterns shared between ischemia-reperfusion and hibernation is the high-low-low pattern **(A** and **B**, top left**)**. One major difference between ischemia-reperfusion and hibernation is that hibernation exhibits a low-high-high pattern **(B**, right bottom**)**. CN: control; IS: ischemia; RP: reperfusion; T: torpor; A: arousal episodes; P: post-reproduction.

To compare the overall trend of gene expression in ischemia-reperfusion and hibernation, self-organizing mapping was performed for 4,408 overlapping genes (Figure 3). One of the major patterns shared between ischemia-reperfusion and hibernation is the high-low-low pattern in CN-IS-RP (Figure 3A, top left) and P-T-A (Figure 3B, top left) comparisons. For the > 70% of genes exhibiting these patterns, the expression trends were very similar between ischemia-reperfusion and hibernation, as indicated by the positive correlation (PCC > 0.9) of log2 fold-changes between pair-wise comparisons in CN-IS-RP and P-T-A.

**Figure 4:**
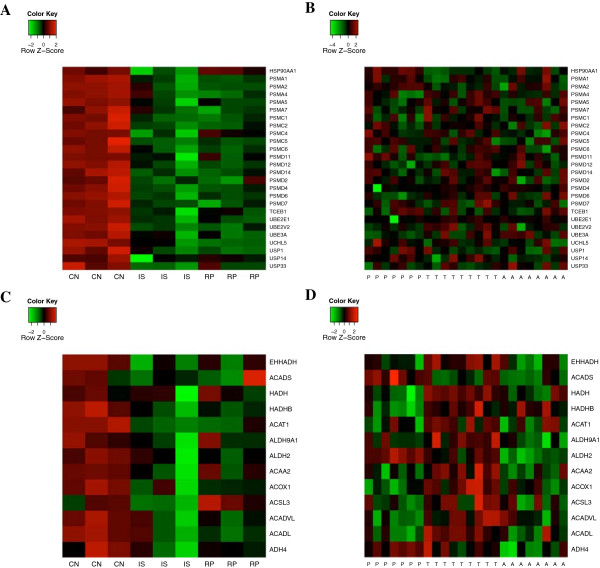
**Heat maps of genes in the protein-ubiquitination pathway and fatty acid metabolism during ischemia-reperfusion and hibernation.** Genes involved in the protein-ubiquitination pathway decrease in expression during ischemia-reperfusion **(A)** but not during hibernation **(B)**. Genes involved in fatty acid metabolism displayed lower expressions in ischemia-reperfusion **(C)** but greater expressions in torpor than in either arousal episodes or post-reproduction **(D)**. CN: control; IS: ischemia; RP: reperfusion; T: torpor; A: arousal episodes; P: post-reproduction.

The under-expression of genes in the protein-ubiquitination pathway leads to severe ischemia-reperfusion injury [42]. These genes decreased in expression during ischemia-reperfusion in old mice (Figure 4A). However, they were not affected during hibernation (Figure 4B), indicating protective functions against ischemia-reperfusion during hibernation. One major difference between ischemia-reperfusion and hibernation in the self-organizing map is that hibernation displays a pattern of low-high-high in P-T-A of hibernation (Figure 3B, bottom right). In this pattern unique to hibernation, expression levels of these genes are elevated during the hibernation season in both T and A when compared with P. They are enriched in fatty acid β-oxidation and PPAR signaling pathways. We further examined the expression of the 13 genes (*EHHADH*, *ACADS*, *HADH*, *HADHB*, *ACAT1*, *ALDH9A1*, *ALDH2*, *ACAA2*, *ACOX1*, *ACSL3*, *ACADL*, and *ADH4*) involved in fatty acid metabolism. They had lower expression in IS and RP compared with CN in the ischemia-reperfusion dataset but greater expression in T compared with A and P in the hibernation dataset (Figure 4C, 4D). Therefore, the protective functions of hibernation against ischemia-reperfusion might be derived from the increase in fatty acid metabolism.

### Summary

**Figure 5:**
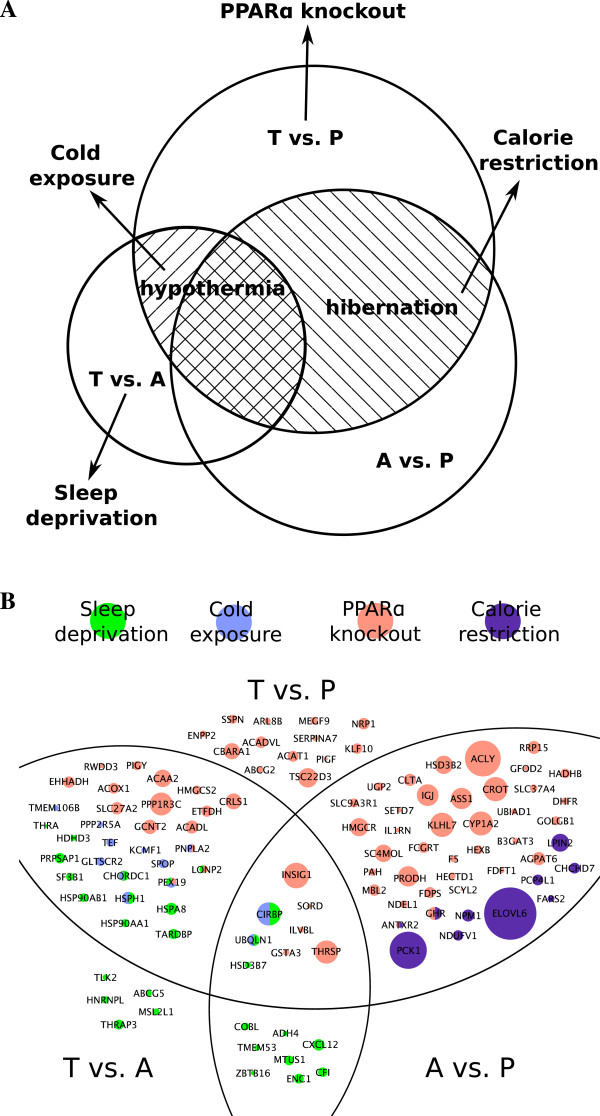
**Comparison of molecular signatures during hibernation to non-hibernation physiological conditions. (A)** The most significant molecular signatures during hibernation in comparison with calorie restriction, PPARα knockout, sleep deprivation, and cold exposure. **(B)** Highlighted genes exhibited reasonable changes in direction in physiological conditions and hibernation (opposite in PPARα knockout and T vs. P, opposite in sleep deprivation and T vs. A, consistent in calorie restriction and hibernation signatures, consistent in cold exposure and hypothermia signatures). The arc lines divide the different hibernation signatures (T vs. P, T vs. A, and A vs. P). The color of the gene indicates the condition it belongs to. Genes with multiple colors are differentially expressed in more than one physiological condition. The gene sizes were scaled to log2 fold-changes of genes in hibernation. T: torpor; A: arousal episodes; P: post-reproduction.

The most significant molecular signatures of hibernation when compared with non-hibernating physiological phenotypes are shown in Figure 5A. These include comparisons of PPARα knockout to TP signatures, sleep deprivation to TA signatures, calorie restriction to hibernation signatures, and cold exposure to hypothermia signatures. The genes involved in these comparisons are shown in Figure 5B.

## Discussion

### Metabolic shifts during hibernation

On the physiological level, similarities between fasting during hibernation and calorie restriction are manifested in several ways, for instance, blood glucose and blood insulin [43]. Fasting mice are prone to lower body temperatures and enter a torpor-like state. Calorie restriction in mice and hibernation in the AGS shared many molecular signatures involved in shifts in metabolic fuel use, e.g. high expression of gluconeogenesis and low expression of fatty acid biosynthesis, together with regulatory changes to suppress cell growth. The results from the PPARα knockout and ChIP-Seq experiments in combination provide further support that PPARα is a key regulator that mediates the switch in metabolism from carbohydrates to fatty acids during torpor.

### Sleep-wake and torpor-arousal cycles

In the circadian cycle the Tb of most animals, including mice, oscillates. Tb is high when mice are awake and lower when they are sleeping. During hibernation, core AGS Tb remains close to 0°C for over 3 weeks during torpor and returns to euthermic levels for less than 16 h during arousal episodes. Such similarities have led to the hypothesis that the torpor-arousal cycle is the result of the expression of peripheral clocks, but not the central circadian clock, that persists in a non-temperature compensated manner during hibernation [44]. Our results support this hypothesis based on the expression changes of temperature-responsive genes, e.g. heat shock protein genes and *CIRBP*. Therefore, these temperature-responsive genes may be components of a non-temperature compensated peripheral clock. Recently, the circadian oscillation of Tb controlled by the SCN was proposed to serve as a global entrainment cue to synchronize peripheral clocks, in which the heat shock pathway was shown to play a critical role [45,46]. *CIRBP* modulates circadian gene expression post-transcriptionally [28]. Our analysis suggests that *CIRBP* directly regulates several other transcription factors and RNA-binding proteins that are potentially important during hibernation. In combination, heat shock proteins and *CIRBP* may be important links between the torpor-arousal cycle during hibernation and sleep-wake and activity cycles in circadian rhythms.

### Ischemia-reperfusion and hibernation

Whether cycles of torpor and arousal and ischemia-reperfusion represent similar physiological conditions is controversial. Blood flow, though vastly reduced, continues during steady-state torpor and meets a reduced metabolic demand. During arousal, however, blood pressure and flow quickly rise with evidence of oxidative stress [47]. We identified a large set of genes with strong low-high-high patterns comparing torpor, arousal, and post-hibernation that occurred during hibernation but was absent in mouse ischemia-reperfusion, in which fatty acid β-oxidation and PPAR signaling associated genes are over-represented. The protective effects of long-chain polyunsaturated fatty acids on ischemia-reperfusion injury have been shown in the rat heart [48] and liver [49]. Our study implies that the increase in fatty acid metabolism might protect against ischemia-reperfusion injury.

## Conclusions

This is the first genome-wide gene expression study to systematically compare stages in the hibernation phenotype with gene expression patterns in calorie restriction, sleep deprivation, cold exposure, circadian cycles, and ischemia-reperfusion in mice. Two important regulators, PPARα and *CIRBP*, have been examined for their roles in metabolic shift and hypothermia during hibernation. Undoubtedly, hibernation as an integrated and complex phenotype differs from each of the physiological conditions that we compared. There is only limited overlap of molecular signatures between hibernation and the physiological conditions that we examined in mice. Some of the differences we observed may be due to species differences between the AGS and mice. Another major difference between hibernation in the AGS and the conditions in mice is Tb. Transcription and translation are greatly decreased in the near zero AGS Tb during hibernation but not in any of the physiological conditions we examined in mice. The over-expressed mRNA transcripts in torpor may have been elevated prior to entry or re-entry into torpor. These transcripts may be translated into proteins during arousal episodes to fulfill their functions during torpor. Nevertheless, the shared molecular signatures identified in our study will further our understanding of the relationship between hibernation and alternative phenomena in non-hibernating animals and promote its application in human medicine.

## Abbreviations

AGS: Arctic ground squirrel; Tb: Body temperature; EEG: Electroencephalogram; A: Arousal episodes; T: Torpor; P: Post-reproduction; IS: Ischemia; RP: Reperfusion; CN: Control; EST: Expressed sequence tag; HSD: Honestly significant difference; FDR: False discovery rate; PCC: Pearson’s correlation coefficient; ZT: Zeitgeber time; ChIP-Seq: Chromatin immunoprecipitation with massively parallel DNA sequencing; CLIP-Seq: Cross-linking and immunoprecipitation-high-throughput sequencing.

## Competing interests

The authors declare that they have no conflicts of interest.

## Authors’ contributions

JY conceived the study. AVG and VBF collected the samples and conducted the expression experiments. CS designed and annotated microarray probes. YX and JY analyzed the data. YX, JY, BMB, and VBF wrote the paper. All authors read and approved the final manuscript.

## Supplementary Material

Additional file 1: Table S1 ANOVA results of differentially expressed genes during hibernation. Probe ID indicates the probe ID on the microarray. Gene indicates the annotation of probes in the human gene symbol. ANOVA p indicates p-values of ANOVA between post-reproduction (P), torpor (T), and arousal episodes (A). The 4th column showes q-values of ANOVA p-values after multiple test correction. The 5th-7th columns show log2 transformed signals in A, P, and T states, respectively. The 8-10th columns show log2 fold-changes (LFC) between each pair of states. The 11-13th columns show Tukey’s honestly significant difference test p-value between each pair of states. The rows were ranked in the order of the q-value. Click here for file

Additional file 2: Table S2 Meta-analysis results of differentially expressed genes during calorie restriction. The calorie restriction column is the mean of log2 fold-changes between calorie restriction and control in the meta-analysis. Click here for file

Additional file 3: Table S3 Genes overlapping between certain parts of the hibernation signatures and corresponding physiological conditions. The 2-4th columns show the log2 fold-changes of gene expression in TP, AP, and TA signatures. The 5-8th columns show the log2 fold-changes of gene expression during calorie restriction, PPARα knockout, sleep deprivation, and cold exposure, when compared with hibernation, TP, TA, and hypothermia signatures, respectively. The 9th column shows the phases of circadian genes in TA signatures. The T in the 10th column indicates the PPARα target gene in TP signatures. The symbol - indicates the gene is not available or significant to the corresponding condition. TP: torpor vs. post-reproduction; AP: arousal episodes vs. post-reproduction; TA: torpor vs. arousal episodes. Click here for file

## References

[B1] Barnes B Freeze avoidance in a mammal: body temperatures below 0 degree C in an Arctic hibernator Science 1989 244 1593 1595 10.1126/science.2740905 2740905

[B2] van Breukelen F Martin SL Reversible depression of transcription during hibernation Journal of comparative physiology. B, Biochemical, systemic, and environmental physiology 2002 172 355 61 10.1007/s00360-002-0256-1 12122451

[B3] Frerichs K Smith C Brenner M Degracia D Krause G Marrone L Dever T Hallenbeck J Suppression of protein synthesis in brain during hibernation involves inhibition of protein initiation and elongation Proc Natl Acad Sci U S A 1998 95 14511 14516 10.1073/pnas.95.24.14511 9826731 PMC24404

[B4] Carey HV Andrews MT Martin SL Mammalian hibernation: cellular and molecular responses to depressed metabolism and low temperature Physiological reviews 2003 83 1153 81 14506303 10.1152/physrev.00008.2003

[B5] Heller HC Ruby NF Sleep and circadian rhythms in mammalian torpor Annual review of physiology 2004 66 275 89 10.1146/annurev.physiol.66.032102.115313 14977404

[B6] Trachsel L Edgar DM Heller HC Are ground-squirrels sleep-deprived during hibernation Am J Physiol 1991 260 R1123 R1129 2058740 10.1152/ajpregu.1991.260.6.R1123

[B7] Daan S Barnes B Warming up for sleep? Neurosci Lett 1991 128 265 268 10.1016/0304-3940(91)90276-Y 1945046

[B8] Epperson LE Karimpour-Fard A Hunter LE Martin SL Metabolic cycles in a circannual hibernator Physiological genomics 2011 43 799 807 10.1152/physiolgenomics.00028.2011 21540299 PMC3132838

[B9] Revel FG Herwig A Garidou M-L Dardente H Menet JS Masson-Pévet M Simonneaux V Saboureau M Pévet P The circadian clock stops ticking during deep hibernation in the European hamster Proc Natl Acad Sci U S A 2007 104 13816 20 10.1073/pnas.0704699104 17715068 PMC1959465

[B10] Hut R Barnes B Daan S Body temperature patterns before, during, and after semi-natural hibernation in the European ground squirrel Journal of Comparative Physiology B: Biochemical, Systemic, and Environmental Physiology 2002 172 47 58 10.1007/s003600100226 11824403

[B11] Williams CT Barnes BM Buck CL Daily body temperature rhythms persist under the midnight sun but are absent during hibernation in free-living arctic ground squirrels Biology letters 2012 8 31 4 10.1098/rsbl.2011.0435 21752811 PMC3259947

[B12] Lindell SL Klahn SL Piazza TM Mangino MJ Torrealba JR Southard JH Carey HV Natural resistance to liver cold ischemia-reperfusion injury associated with the hibernation phenotype American journal of physiology. Gastrointestinal and liver physiology 2005 288 G473 80 15701622 10.1152/ajpgi.00223.2004

[B13] Kurtz CC Lindell SL Mangino MJ Carey HV Hibernation confers resistance to intestinal ischemia-reperfusion injury American journal of physiology. Gastrointestinal and liver physiology 2006 291 G895 901 10.1152/ajpgi.00155.2006 16751173

[B14] Podgoreanu MV Ma Q Mackensen GB Zhang Z Smith MP Bain J Newgard CB Kohl F Drew KL Barnes BM The hibernator metabolic phenotype is cardioprotective in the setting of deep hypothermic circulatory arrest [abstract] Circulation 2011 124 A17046

[B15] Drew KL Buck CL Barnes BM Christian SL Rasley BT Harris MB Central nervous system regulation of mammalian hibernation: implications for metabolic suppression and ischemia tolerance Journal of neurochemistry 2007 102 1713 26 10.1111/j.1471-4159.2007.04675.x 17555547 PMC3600610

[B16] Subramanian A Tamayo P Mootha VK Mukherjee S Ebert BL Gillette M Paulovich A Pomeroy SL Golub TR Lander ES Mesirov JP Gene set enrichment analysis: a knowledge-based approach for interpreting genome-wide expression profiles Proc Natl Acad Sci U S A 2005 102 15545 50 10.1073/pnas.0506580102 16199517 PMC1239896

[B17] Yan J Barnes BM Kohl F Marr TG Modulation of gene expression in hibernating arctic ground squirrels Physiological genomics 2008 32 170 81 17925484 10.1152/physiolgenomics.00075.2007

[B18] Yan J Burman A Nichols C Alila L Showe LC Showe MK Boyer BB Barnes BM Marr TG Detection of differential gene expression in brown adipose tissue of hibernating arctic ground squirrels with mouse microarrays Physiological genomics 2006 25 346 53 10.1152/physiolgenomics.00260.2005 16464973

[B19] Buck CL Barnes BM Effects of ambient temperature on metabolic rate, respiratory quotient, and torpor in an arctic hibernator American journal of physiology. Regulatory, integrative and comparative physiology 2000 279 R255 62 10896889 10.1152/ajpregu.2000.279.1.R255

[B20] Zhu YY Machleder EM Chenchik A Li R Siebert PD Reverse transcriptase template switching: a SMART approach for full-length cDNA library construction Biotechniques 2001 30 892 7 11314272 10.2144/01304pf02

[B21] Carninci P Normalization and subtraction of cap-trapper-selected cDNAs to prepare full-length cDNA libraries for rapid discovery of new genes Genome Res 2000 10 1617 1630 10.1101/gr.145100 11042159 PMC310980

[B22] Fedorov VB Goropashnaya AV Tøien O Stewart NC Gracey AY Chang C Qin S Pertea G Quackenbush J Showe LC Showe MK Boyer BB Barnes BM Elevated expression of protein biosynthesis genes in liver and muscle of hibernating black bears (Ursus americanus) Physiological genomics 2009 37 108 18 10.1152/physiolgenomics.90398.2008 19240299 PMC3774579

[B23] Storey JD Tibshirani R Statistical significance for genomewide studies Proc Natl Acad Sci U S A 2003 100 9440 5 10.1073/pnas.1530509100 12883005 PMC170937

[B24] Rakhshandehroo M Sanderson LM Matilainen M Stienstra R Carlberg C de Groot PJ Müller M Kersten S Comprehensive analysis of PPARalpha-dependent regulation of hepatic lipid metabolism by expression profiling PPAR research 2007 2007 26839 18288265 10.1155/2007/26839 PMC2233741

[B25] de Magalhães JP Curado J Church GM Meta-analysis of age-related gene expression profiles identifies common signatures of aging Bioinformatics 2009 25 875 81 10.1093/bioinformatics/btp073 19189975 PMC2732303

[B26] Tamayo P Slonim D Mesirov J Zhu Q Kitareewan S Dmitrovsky E Lander ES Golub TR Interpreting patterns of gene expression with self-organizing maps: methods and application to hematopoietic differentiation Proc Natl Acad Sci U S A 1999 96 2907 12 10.1073/pnas.96.6.2907 10077610 PMC15868

[B27] Boergesen M Pedersen TÅ Gross B van Heeringen SJ Hagenbeek D Bindesbøll C Caron S Lalloyer F Steffensen KR Nebb HI Gustafsson J-Å Stunnenberg HG Staels B Mandrup S Genome-wide profiling of liver X receptor, retinoid X receptor, and peroxisome proliferator-activated receptor α in mouse liver reveals extensive sharing of binding sites Molecular and cellular biology 2012 32 852 67 10.1128/MCB.06175-11 22158963 PMC3272984

[B28] Morf J Rey G Schneider K Stratmann M Fujita J Naef F Schibler U Cold-inducible RNA-binding protein modulates circadian gene expression posttranscriptionally Science 2012 338 379 83 10.1126/science.1217726 22923437

[B29] Florant GL Healy JE The regulation of food intake in mammalian hibernators: a review Journal of comparative physiology. B, Biochemical, systemic, and environmental physiology 2012 182 451 467 10.1007/s00360-011-0630-y 22080368

[B30] Tsuchiya T Dhahbi JM Cui X Mote PL Bartke A Spindler SR Additive regulation of hepatic gene expression by dwarfism and caloric restriction Physiological genomics 2004 17 307 15 10.1152/physiolgenomics.00039.2004 15039484

[B31] Dhahbi JM Mote PL Fahy GM Spindler SR Identification of potential caloric restriction mimetics by microarray profiling Physiological genomics 2005 23 343 50 10.1152/physiolgenomics.00069.2005 16189280

[B32] Selman C Kerrison ND Cooray A Piper MDW Lingard SJ Barton RH Schuster EF Blanc E Gems D Nicholson JK Thornton JM Partridge L Withers DJ Coordinated multitissue transcriptional and plasma metabonomic profiles following acute caloric restriction in mice Physiological genomics 2006 27 187 200 10.1152/physiolgenomics.00084.2006 16882887

[B33] Gropler MC Harris TE Hall AM Wolins NE Gross RW Han X Chen Z Finck BN Lipin 2 is a liver-enriched phosphatidate phosphohydrolase enzyme that is dynamically regulated by fasting and obesity in mice The Journal of biological chemistry 2009 284 6763 72 19136718 10.1074/jbc.M807882200 PMC2652272

[B34] Burton KA White P Harrison AP Gilmour RS Duchamp C Nutritional D Nutritional expression regulation of growth hormone receptor FASEB J 1994 8 81 88 7507871 10.1096/fasebj.8.1.7507871

[B35] Reddy JK Hashimoto T Peroxisomal beta-oxidation and peroxisome proliferator-activated receptor alpha: an adaptive metabolic system Annu Rev Nutr 2001 21 193 230 10.1146/annurev.nutr.21.1.193 11375435

[B36] Maret S Dorsaz S Gurcel L Pradervand S Petit B Pfister C Hagenbuchle O O’Hara BF Franken P Tafti M Homer1a is a core brain molecular correlate of sleep loss Proc Natl Acad Sci U S A 2007 104 20090 5 10.1073/pnas.0710131104 18077435 PMC2148427

[B37] Imada S Yamamoto M Tanaka K Seiwa C Watanabe K Kamei Y Kozuma S Taketani Y Hypothermia-induced increase of Oligodendrocyte precursor cells : possible involvement of Plasmalemmal voltage-dependent Anion channel 1 J Neurosci Res 2010 3466 3457 3466 20936704 10.1002/jnr.22520

[B38] Gano JJ Simon J a A proteomic investigation of ligand-dependent HSP90 complexes reveals CHORDC1 as a novel ADP-dependent HSP90-interacting protein Molecular & cellular proteomics : MCP 2010 9 255 70 10.1074/mcp.M900261-MCP200 19875381 PMC2830838

[B39] Yan J Wang H Liu Y Shao C Analysis of gene regulatory networks in the mammalian circadian rhythm PLoS computational biology 2008 4 e1000193 10.1371/journal.pcbi.1000193 18846204 PMC2543109

[B40] Al-Fageeh MB Smales CM Control and regulation of the cellular responses to cold shock: the responses in yeast and mammalian systems The Biochemical journal 2006 397 247 59 10.1042/BJ20060166 16792527 PMC1513281

[B41] Warraich ST Yang S Nicholson G a Blair IP TDP-43: a DNA and RNA binding protein with roles in neurodegenerative diseases The international journal of biochemistry & cell biology 2010 42 1606 9 10.1016/j.biocel.2010.06.016 20601083

[B42] Huber N Sakai N Eismann T Shin T Kuboki S Blanchard J Schuster R Edwards MJ Wong HR Lentsch AB Age-related decrease in proteasome expression contributes to defective nuclear factor-kappaB activation during hepatic ischemia/reperfusion Hepatology 2009 49 1718 28 10.1002/hep.22840 19206148 PMC2695826

[B43] Walford RL Spindler SR The response to calorie restriction in mammals shows features also common to hibernation: a cross-adaptation hypothesis The journals of gerontology. Series A, Biological sciences and medical sciences 1997 52 B179 83 9224421 10.1093/gerona/52a.4.b179

[B44] Malan A Is the torpor-arousal cycle of Hibernation controlled by a non-temperature-compensated circadian clock? Journal of biological rhythms 2010 25 166 175 10.1177/0748730410368621 20484688

[B45] Abraham U Granada AE Westermark PO Heine M Kramer A Herzel H Coupling governs entrainment range of circadian clocks Molecular systems biology 2010 6 438 21119632 10.1038/msb.2010.92 PMC3010105

[B46] Buhr ED Yoo S-H Takahashi JS Temperature as a universal resetting cue for mammalian circadian oscillators Science 2010 330 379 85 10.1126/science.1195262 20947768 PMC3625727

[B47] Drew KL Rice ME Kuhn TB Smith MA Neuroprotective adaptations in hibernation: therapeutic implications for ischemia-reperfusion, traumatic brain injury and neurodegenerative diseases Free Radic Biol Med 2001 31 563 573 10.1016/S0891-5849(01)00628-1 11522441

[B48] Engelbrecht A-M Engelbrecht P Genade S Niesler C Page C Smuts M Lochner A Long-chain polyunsaturated fatty acids protect the heart against ischemia/reperfusion-induced injury via a MAPK dependent pathway Journal of molecular and cellular cardiology 2005 39 940 54 10.1016/j.yjmcc.2005.08.004 16216266

[B49] Zúñiga J Venegas F Villarreal M Núñez D Chandía M Valenzuela R Tapia G Varela P Videla L a Fernández V Protection against in vivo liver ischemia-reperfusion injury by n-3 long-chain polyunsaturated fatty acids in the rat Free radical research 2010 44 854 63 10.3109/10715762.2010.485995 20528561

